# Changes in neuronal excitability and synaptic transmission in nucleus accumbens in a transgenic Alzheimer’s disease mouse model

**DOI:** 10.1038/s41598-020-76456-w

**Published:** 2020-11-11

**Authors:** E. J. Fernández-Pérez, S. Gallegos, L. Armijo-Weingart, A. Araya, N. O. Riffo-Lepe, F. Cayuman, L. G. Aguayo

**Affiliations:** Laboratory of Neurophysiology, Department of Physiology, Universidad de Concepción, Barrio Universitario S/N, P. O. Box 160-C, Concepción, Chile

**Keywords:** Cellular neuroscience, Diseases of the nervous system

## Abstract

Several previous studies showed that hippocampus and cortex are affected in Alzheimer’s disease (AD). However, other brain regions have also been found to be affected and could contribute with new critical information to the pathophysiological basis of AD. For example, volumetric studies in humans have shown a significant atrophy of the striatum, particularly in the nucleus Accumbens (nAc). The nAc is a key component of the limbic reward system and it is involved in cognition and emotional behaviors such as pleasure, fear, aggression and motivations, all of which are affected in neurodegenerative diseases such as AD. However, its role in AD has not been extensively studied. Therefore, using an AD mouse model, we investigated if the nAc was affected in 6 months old transgenic 2xTg (APP/PS1) mice. Immunohistochemistry (IHC) analysis in 2xTg mice showed increased intraneuronal Aβ accumulation, as well as occasional extracellular amyloid deposits detected through Thioflavin-S staining. Interestingly, the intracellular Aβ pathology was associated to an increase in membrane excitability in dissociated medium spiny neurons (MSNs) of the nAc. IHC and western blot analyses showed a decrease in glycine receptors (GlyR) together with a reduction in the pre- and post-synaptic markers SV2 and gephyrin, respectively, which correlated with a decrease in glycinergic miniature synaptic currents in nAc brain slices. Additionally, voltage-clamp recordings in dissociated MSNs showed a decrease in AMPA- and Gly-evoked currents. Overall, these results showed intracellular Aβ accumulation together with an increase in excitability and synaptic alterations in this mouse model. These findings provide new information that might help to explain changes in motivation, anhedonia, and learning in the onset of AD pathogenesis.

## Introduction

One of the main pathological hallmarks of Alzheimer’s disease (AD) is the presence of aggregates of amyloid beta (Aβ) in brain parenchyma, which has been extensively reported in the hippocampus and cortex in AD brains^1,2^ and in several mice models of the disease^3–7^. Interestingly, the presence of Aβ is not restricted to these regions important for memory and cognition since studies have reported pathological Aβ accumulation in the limbic system, particularly in the striatum in humans^8,9^. In addition, volumetric brain studies have shown significant atrophy of limbic regions, and the nucleus accumbens (nAc) appears to be largely affected^10^, suggesting a role for this region in the disease progression. The nAc is a critical region in the brain reward system^11^ and together with other mesolimbic regions plays a role in the neuronal processing of natural rewarding stimuli such as food and water ingestion, and sexual activity^12^. In addition, it is well accepted that drugs of abuse are also able to activate this subcortical circuitry^11,13,14^.

Medium spiny neurons (MSNs) are the principal projecting neurons in the nAc that are inhibitory in nature by releasing GABA at their projecting terminals^12,15^. The control of neuronal excitability within the nAc is significant because it acts as an integrative center that receives inputs from different brain areas. For example, it receives dopaminergic signaling from the ventral tegmental area (VTA) and dense glutamatergic inputs from the prefrontal cortex (PFC), basolateral amygdala, and the ventral subiculum of the hippocampus^11,13,16–19^. All the information converging in the nAc makes this region relevant in terms of “go” and “no-go” learning and action selection by integrating cognitive and affective information^12^. Although the role of the nAc in reward, aversion, addictions, cognition, learning, and emotional behaviors in the normal brain are well recognized^12,20–24^, much less is known about its role in the symptomatology that is associated to neurodegenerative diseases such as AD^25–27^.

Here, we describe the presence of Aβ pathology in the nAc of a double transgenic AD mouse model (APP/PS1; denoted 2xTg) with no difference in neuronal number or astrogliosis. The neurons exhibited an increase in excitability, decreased pre and post synaptic markers, and alterations in postsynaptic currents. Our work suggests that at early stages of the disease, changes in synaptic and membrane excitability properties in the nAc could have important implications for mesolimbic symptoms in AD.

## Methods

### Animals

Animal care and experimental protocols for this study were approved by the Institutional Animal Care and Use Committee at the University of Concepción. 6–7 months old C57BL/6 J and 2xTg transgenic mice (MMRRC034832 B6.Cg­Tg (APPswe,PSEN1dE9)85Dbo/Mmjax) were obtained from Jackson Laboratory (Bar Harbor, ME, USA). The 2xTg mice express the Swedish mutation (K594M/N595L) and a human exon‐9‐deleted variant of PS1 (PS1‐dE9) and secrete higher levels of Aβ^28^. Mice were individually housed in groups of 2–4 on a 12 h light/dark cycle and given food and water ad libitum. Mice were sacrificed by decapitation after anesthesia with isoflurane as described previously^29^.

### Preparation of acute brain slices

Coronal brain slices containing the nAc were prepared as previously described^30^. Immediately after excision the brain was placed in ice-cold cutting solution (in mM): Sucrose 194, NaCl 30, KCl 4.5, MgCl_2_ 1, NaHCO_3_ 26, NaH_2_PO_4_ 1.2, Glucose 10, saturated with 95% O_2_ and 5% CO_2_ and adjusted to pH 7.4. The brain was cut and glued to the chilled stage of a VT1200S vibratome (Leica, Germany) and sliced to a thickness of 300 µm. Slices containing the nAc were transferred to aCSF solution (in mM): NaCl 124, KCl 4.5, MgCl_2_ 1, NaHCO_3_ 26, NaH_2_PO_4_ 1.2, Glucose 10, CaCl_2_ 2, saturated with 95% O_2_ and 5% CO_2_ at 32 °C and adjusted to pH 7.4 and 315–320 mOsm. Brain slices were allowed to rest in O_2_–perfused aCSF at 32 °C for at least 1 h before recording or enzymatic treatment for dissociation.

### Mechano-chemical dissociation of MSNs

Preparation of acutely isolated neurons was performed as previously described^31^. See details in Supplementary Information, Sect. 1.1.

### Current- and voltage-clamp experiments in dissociated MSNs

To study the membrane potential (Vm), recordings were obtained in current-clamp mode and the following internal solution was used (in mM): 114 Potassium D-gluconate, 4 KCl, 4 MgCl_2_, 10 BAPTA and 10 HEPES (pH 7.4 adjusted with KOH, 290 mOsm/L). Before starting the Vm recording, a small holding current (− 2 to − 50 pA) was applied to stabilize the resting membrane potential (RMP) to − 70 mV. Some recordings were performed without injection of depolarizing current pulses. To evoke action potentials (AP), a family of current pulses applied for 300 ms was used (from  −300 pA to + 275 pA, increasing by 25 pA steps). For details in AP parameters, input resistance and rheobase calculations see Supplementary Information Sect. 1.2. Voltage-clamp recordings in vitro were performed using the same internal solution for current clamp experiments. To record AMPA, GABA, and glycine (Gly) evoked currents, a gravity-driven perfusion system (2–3 ml/min) was used to extracellularly apply AMPA (100 µM), GABA (100 µM), and Gly (1000 µM), respectively. Excitatory currents (AMPA) were recorded by adjusting the membrane potential to − 60 mV and inhibitory currents (GABA and Gly) at 0 mV. Recordings were performed using an Axopatch-200B amplifier (Molecular Devices, USA) and an inverted microscope (Nikon Eclipse TE200-U, Japan). The acquisition was made using a computer connected to the recording system using a Digidata 1440A acquisition card (Molecular Devices, USA) and the pClamp10 software (Molecular Devices, USA). Electrodes with a resistance of 4–5 MΩ were pulled from borosilicate capillaries (WPI, USA) in a horizontal puller (P1000, Sutter Instruments, USA). Evoked currents were expressed as the maximum current divided by cell capacitance. To obtain the capacitance, the membrane charge was calculated by integrating the transient capacitive current after subtracting the pipette capacitance using the Chebyshev algorithm to calculate the tau for the decay of the current with pClamp10 software (Molecular Devices, USA). This value divided by the voltage step used to monitor the capacitive current (5 mV) allowed us to obtain the cell capacitance.

### Voltage-clamp experiments ex vivo

Recording in MSNs were performed as previously described^30^. Acute brain-slices containing the nAc were transferred to the recording chamber with aCSF solution saturated with 95% O_2_ and 5% CO_2_ at 30–32 °C. The slices were observed in a DIC-IR microscope using 10 × and 40 × objectives (Nikon Eclipse FN1, Japan) and perfused with oxygenated aCSF at 2 ml/min at 30–32 °C. Patch pipettes with a resistance of 4–5 MΩ were prepared from filament containing borosilicate micropipettes (World Precision Instruments, USA) using a *P*-1000 micropipette puller (Sutter Instruments, USA). The AMPA, GABA and glycinergic miniature postsynaptic currents (mPSCs) were pharmacologically isolated via bath application of groups of blockers such as the NMDA receptor antagonist, D-2-amino-5-phosphonovalerate (D-APV, 50 µM); the nicotinic acetylcholine receptor antagonist, mecamylamine (10 µM); the AMPA receptor antagonist, 6-Cyano-7-nitroquinoxaline-2,3-dione (CNQX, 10 µM); the GABA_A_ antagonist, bicuculline (10 µM); the glycine receptor antagonist, strychnine (4 µM) and tetrodotoxin (TTX; 500 nM). Currents were displayed and stored on a personal computer using a 1322A Digidata (Axon Instruments, Union City, CA, USA), analyzed with Clampfit 10.1 (Axon Instruments, Union City, CA, USA) and MiniAnalysis 6.0 (Synaptosoft Inc.). We show the analysis of frequency (Hz), decay constant (ms), and amplitude (pA). The decay constant of mPSCs was fitted as a single exponential and both rise and decay-phases were fitted between 10 and 90% of the maximal amplitude. The normalized frequency of events was obtained as a percentage reflecting the ratio between either AMPA, GABA, or glycine pharmacologically isolated miniature currents to the total frequency of synaptic events in the absence of inhibitors.

### Immunohistochemistry (IHC)

Free floating frozen 30 μm thick coronal brain slices were used. For tissue preparation, mice were first anesthetized and then intracardially perfused with warm 0.9% saline solution followed by cold freshly prepared 4% paraformaldehyde (PFA). Brains were dissected and post fixed in the same fixative for 24 h at 4 °C. For cryoprotection, brains were placed in a solution with 30% sucrose for 3–5 days at 4 °C. Next, brains were embedded in Neg-50 (Thermofisher) and placed at − 20 °C for 2–4 h and then at − 80 °C for at least 24 h. Frozen brain tissues were sectioned using the cryostat at the CMA facility (https://www.cmabiobio.cl). For immunostaining, free floating slices were washed with Tris-PO_4_ and permeabilized with Tris-1%BSA and 0.2% Triton X-100. Tissues were incubated overnight with primary antibodies αGlyR (1:300, rabbit, Synaptic Systems, Germany), GFAP (1:200, mouse, Synaptic Systems, Germany), Gephyrin (1:300, mouse, Synaptic Systems, Germany), MOAB-2 (1:200, mouse, Novus Biologicals, USA), SV2 (1:200, rabbit, Synaptic Systems, Germany), NeuN (1:1000, rabbit, Abcam, UK) and anti MAP2 (1:200, guinea pig, Synaptic Systems, Germany). After washes with Tris-1% BSA and 0.2% Triton X-100, slices were incubated for 2 h with anti-mouse, anti-rabbit and anti-guinea pig secondary antibodies (1:200, Santa Cruz, USA). TOPRO (1:800, ThermoFisher, USA) or DAPI (300 nM, Thermofisher, USA) were used for nuclear counterstaining. All stained samples were mounted with DAKO fluorescent mounting medium on glass slides and analyzed under confocal microcopy at the CMA facility. We examined at least 3 brain slices per animal and the fluorescence intensity was quantified using the ‘ImageJ’ (NIH) program. The images of nAc were obtained ventral and medial of the anterior commissure (AC).

### Statistical analysis

Statistical analyses were performed using the two‐tailed paired or unpaired Student’s t-tests (α = 0.05) or the two-tailed Mann–Whitney U-test (α = 0.05) as appropriate, after testing for normality with Shapiro–Wilk test and for homogeneity of variances with Levene’s test. Data with more than two groups or factors were analyzed by two-way ANOVA test (α = 0.05) using Origin 2019 (MA, USA). For percentages, chi-square test was used (α = 0.05). Data are shown as mean ± SEM for normally distributed populations and as median and interquartile ranges (IQR) for non-normally distributed populations. n.s. not significant, *p < 0.05, **p < 0.01, ***p < 0.001.

### Ethics approval

All experimental procedures were according to Institutional Animal Care and Use Committee at the University of Concepción animal research regulations.

## Results

### Presence of amyloid pathology in the nAc of 2xTg mice

**Figure 1:**
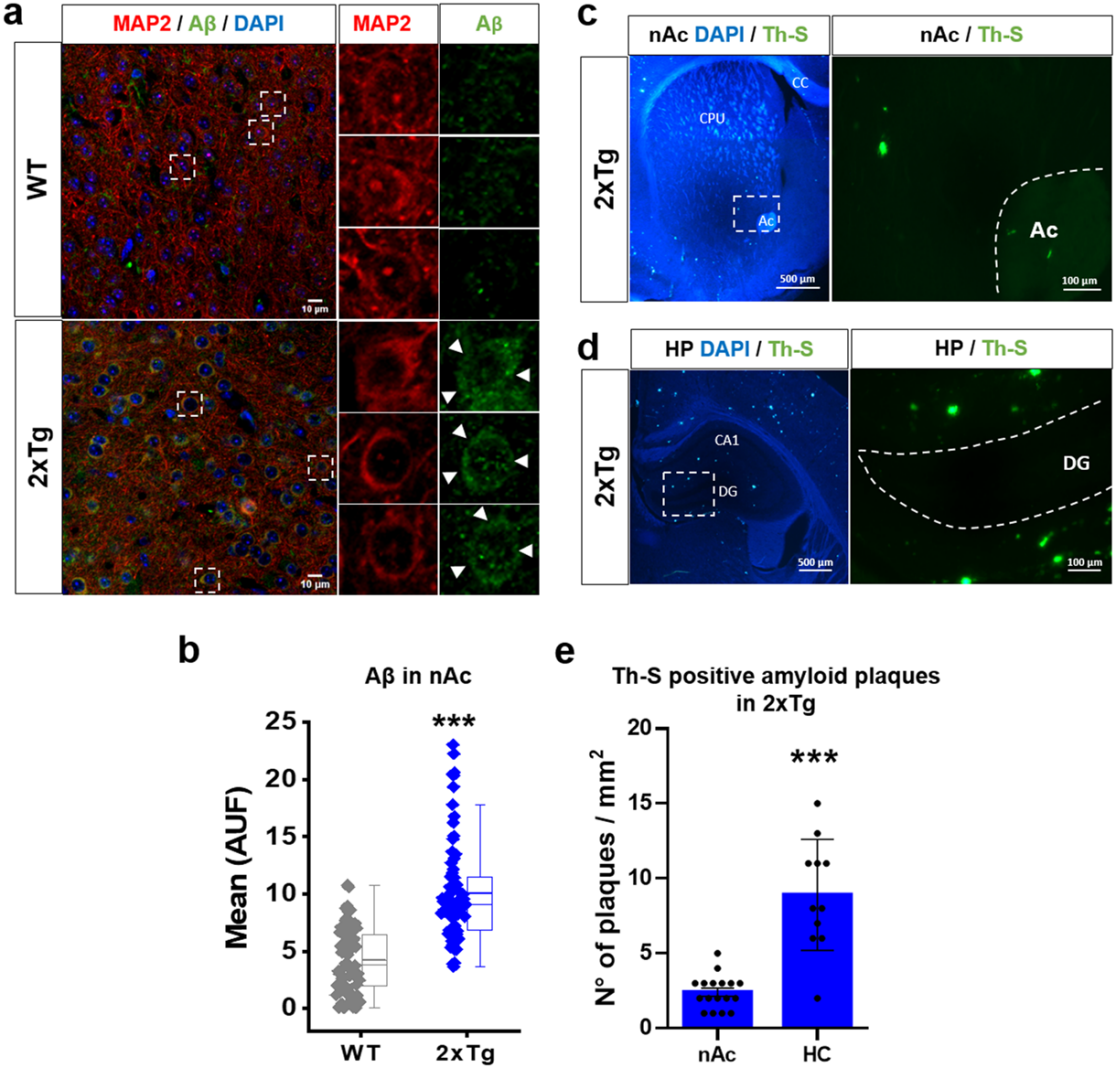
Presence of amyloid pathology in the nAc of 2xTg mouse: (**a**) Immunohistochemistry of coronal slices (30 μm) and high magnification image (inset) showing Aβ in the nAc of 6 months old wild type (WT) and 2xTg mice. Aβ was detected using the MOAB2 antibody. (**b**) Quantification of Aβ fluorescence intensity of WT and 2xTg mice in the soma of nAc neurons (AFU: Arbitrary Fluorescence Units). In 2xTg mice Aβ fluorescence intensity was significantly increased. Data are mean ± SEM and each data point reflects one soma *** *p* < 0.0001. n = 3 WT and 3 2xTg. (**c, d)**, Presence of amyloid plaques stained with Thioflavin S (Th-S) in the nAc (**c**) and compared with hippocampus (HC) (**d**) of 6 months old 2xtg mice (DAPI staining in blue). Inset shows high magnification. CC: corpus callosum; CPU: caudate-putamen; AC: anterior commissure; CA1: subfield of Cornu Ammonis; DG: dentate gyrus. (**e**)**,** Quantification of the number of Th-S-Aβ plaques in nAc and HC. Data are mean ± SEM. (t(11.20) = 5.658, p = 0.0001). n = 3 WT and 3 2xTg. Images of nAc are located ventral and medial to the anterior commissure (not shown). Mann–Whitney test for (**b**), unpaired Student’s t-test with Welch correction for (**e**). Images were analyzed with ‘ImageJ’ 1.8.0_112 (NIH), https://imagej.nih.gov/ij.

To study amyloid pathology in the nAc of WT and 2xTg mice, we first performed an immunohistochemical analysis using a C-terminal targeting antibody for Aβ (see details in methods “Immunohistochemistry (IHC)” section). In WT animals, it was possible to observe a small and diffused presence of Aβ signal in all nAc sections, both in the neuronal processes and soma. In contrast, 2xTg mice showed a substantial increase in Aβ immunodetection, particularly in the intracellular compartment of the neuronal somata in all nAc cells (Fig. 1a, white arrowheads). After quantifying the fluorescence in this region, we found a significant increase of about 2 times with respect to the signal in WT (Fig. 1b). Using Th-S staining, we also found occasional parenchymal amyloid deposits in nAc and hippocampus (HP) but they were detected more frequently in the latter (Fig. 1c–e). No Th-S staining was observed in WT mice (Supplementary Fig. 1). A classical sign of amyloid pathology is the presence of astrogliosis characterized by astrocyte proliferation and overexpression of GFAP (Glial fibrillary acidic protein) intermediate filaments^1^. We did not find any astrogliosis as the presence of GFAP + astrocytes were scarce in 2xTg mice, only 1–2 astrocytes in one of ten slices of nAc of 3 2xTg mice (Supplementary Fig. 2). We did not detect the presence of GFAP + astrocytes in the nAc parenchyma of WT mice (Supplementary Fig. 2). Finally, using NeuN as an immunohistochemical method to identify neuronal cell bodies in histological preparations, we did not find any difference in the number of nAc neurons between the two groups of mice (Supplementary Fig. 2).

**Figure 2:**
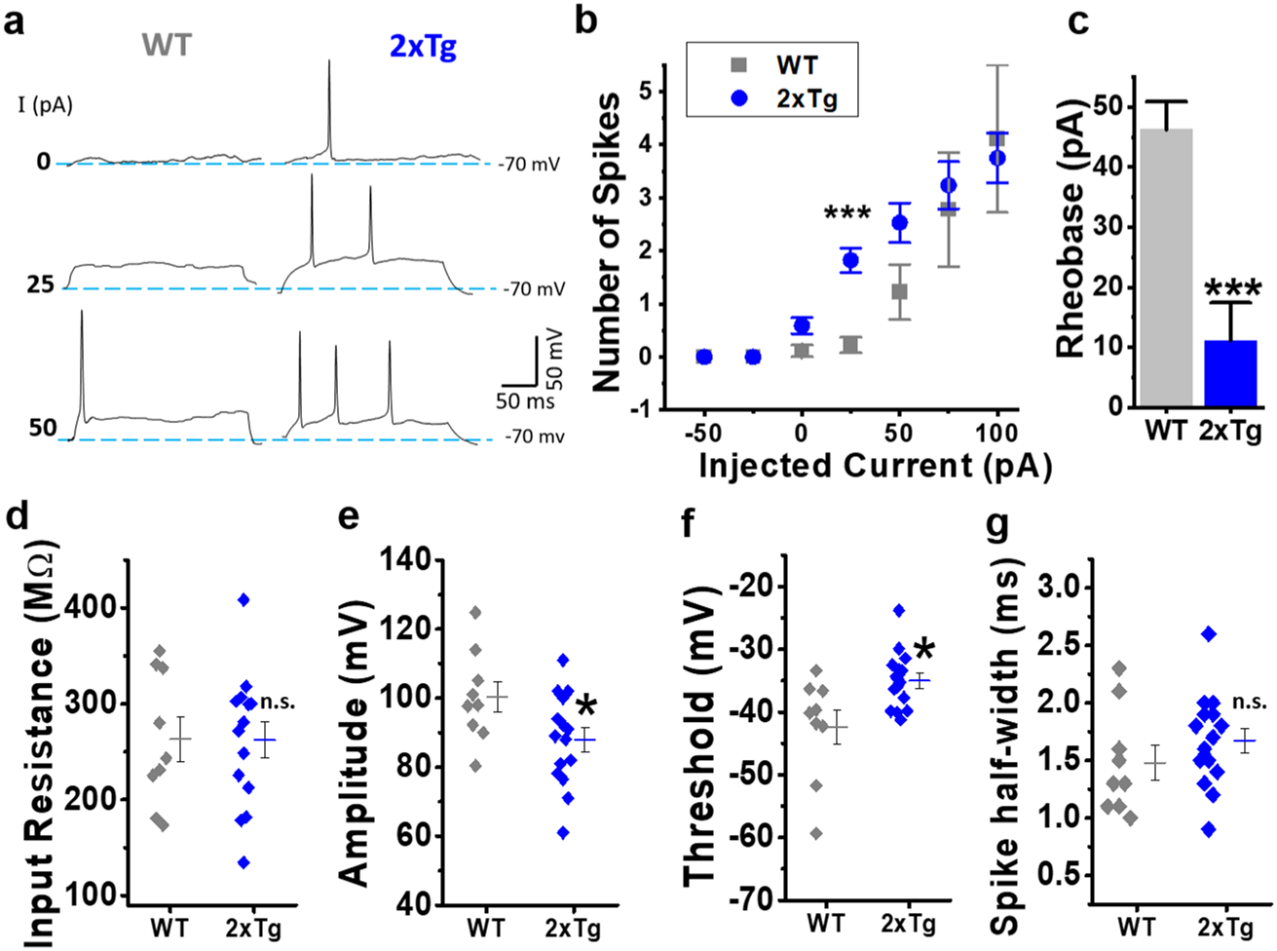
Intrinsic membrane properties of nAc dissociated neurons in WT and 2xTg mice. (**a**) Representative traces of current-clamp recordings after injecting 0, 25 and 50 pA current steps for WT (grey) and 2xTg mice (blue). (**b**) Relationship between the number of spikes generated and the injected current intensity for both conditions (F(1,123) = 12.1639, p = 1.38E-9; *post-hoc* test: with 25 pA, WT vs. 2xTg, p = 1.30E-3; with 50 pA, WT vs 2xTg, p = 7.5E-3). (**c**) Quantification of Rheobase constant (t(22) = 5.475, p = 1.67E-4), (**d**) input resistance (t(22) = 0.029, p = 0.97684) and action potential parameters: (**e**) amplitude (t(22) = 2.226, p = 0.036),(**f**) threshold (t(22) = 2.835, p = 0.009) and (**g**) half-width (t(22) = -0.894, p = 0.380). Scatter and bars graphs represent the average ± SEM. *denotes p < 0.05, **p < 0.01, *** *p* < 0.001 (unpaired Student’s t-test for (**c**–**f)** and (**g**) and two-way ANOVA with Holm-Sidak’s *post-hoc* test for (**b**). Number of neurons: WT = 9 and 2xTg = 15. Neurons obtained in at least 4 independent experiments. Number of mice: WT = 5 and 2xTg = 4.

### Increased intrinsic membrane excitability of nAc MSN in 2xTg mice

The above results suggest an AD-like pathology mainly characterized by the presence of intracellular Aβ accumulation in the nAc. Increased neuronal excitability in neurons with intracellular presence of Aβ has been previously described in the hippocampus^32^, but there are no studies in the nAc. Therefore, we decided to study the intrinsic membrane excitability properties in an in vitro model using acutely dissociated MSNs of nAc from WT and 2xtg mice. The first approach to assess the membrane excitability was to record current-evoked action potential (AP) firing. Our results showed that MSNs of 2xTg exhibited increased membrane excitability since they had a higher number of spikes with lower current stimuli (Fig. 2a). This is clearly observed when plotting spike number vs. injected current that showed a statistical difference between WT and 2xTg (Fig. 2b). The rheobase constant also showed a significant decrease in the 2xTg mice (WT: 46.28 ± 4.61 pA vs.2xTg: 11.14 ± 6.32) (Fig. 2c). On the other hand, the values for input resistance were very similar in both experimental groups (Fig. 2d). The 2xTg mice showed differences in AP amplitude (Fig. 2e) and in AP threshold, which moved towards more depolarized values (Fig. 2f). No changes for AP duration (measured as spike half-width) were found (Fig. 2g).

**Figure 3:**
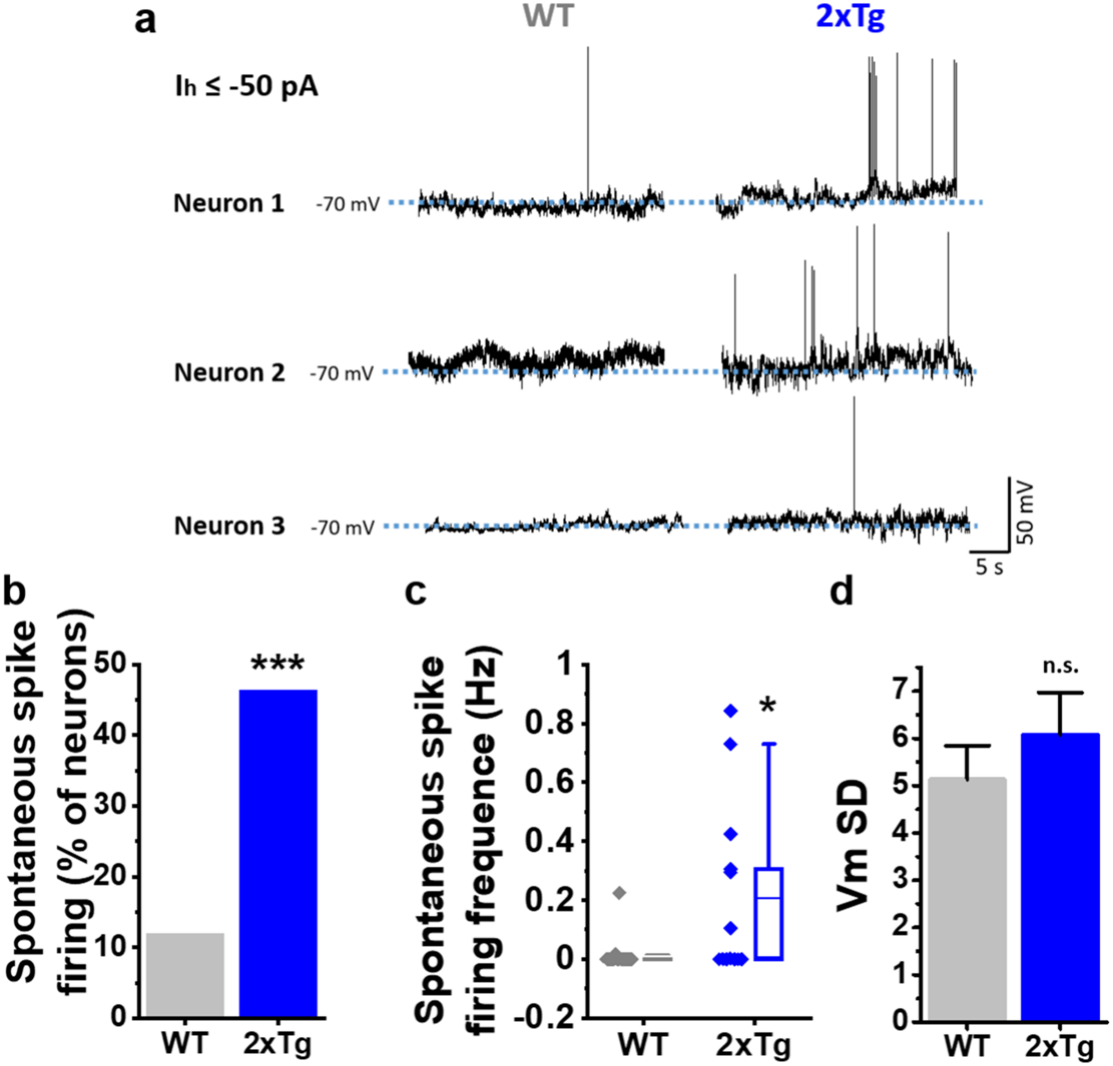
Alterations of spontaneous excitability in isolated nAc neurons in 2xTg mice. (**a**) Membrane potential (Vm) recordings of 3 representative neurons for WT (grey) and 2xTg (blue). (**b**) Percentage of cells that exhibited spontaneous overshooting spikes during the recordings (χ^2^(1,30) = 6.211, *p* = 0.126). (**c**) Quantification of firing frequency of neurons that exhibited spontaneous spikes (U = 60, z-score = − 2.092, *p* = 0.0366). (**d**) Average Vm SD values for the different experimental conditions (t(28) = − 0.840, *p* = 0.407). Bar graphs represent the average ± SEM. *denotes p < 0.05 and ***p < 0.001 (Chi-square test for (**b**), Mann–Whitney-test for (**c**) and unpaired Student’s t-test for (**d**). Number of cells: WT = 17 and 2xTg = 13. Neurons obtained in at least 4 independent experiments. Number of mice: WT = 5 and 2xTg = 4.

The second approach to assess membrane excitability was to record membrane potential (Vm) oscillations without injection of depolarizing current pulses after stabilizing the resting membrane potential (RMP) to − 70 mV (see details in methods, Sect. “Current- and Voltage-clamp experiments in dissociated MSNs”). Under this condition, acutely dissociated MSNs from WT exhibited almost no changes in Vm and in rare cases spontaneous spike firing was observed, whereas 2xTg had a higher prevalence of spontaneous overshooting spikes above zero mV (Fig. 3a). Indeed, we quantified the percentage of neurons that exhibited spontaneous spike firing and it augmented more than fourfold in 2xTg mice (Fig. 3b). In neurons presenting this response, the AP firing frequency was also considerably higher than for WT (Fig. 3c). Finally, no changes in the Vm standard deviation (Vm SD) were found indicating that in basal conditions the fluctuations of Vm were very similar for both mice (Fig. 3d).

### Reduced excitatory and inhibitory receptors activation in 2xTg mice

**Figure 4:**
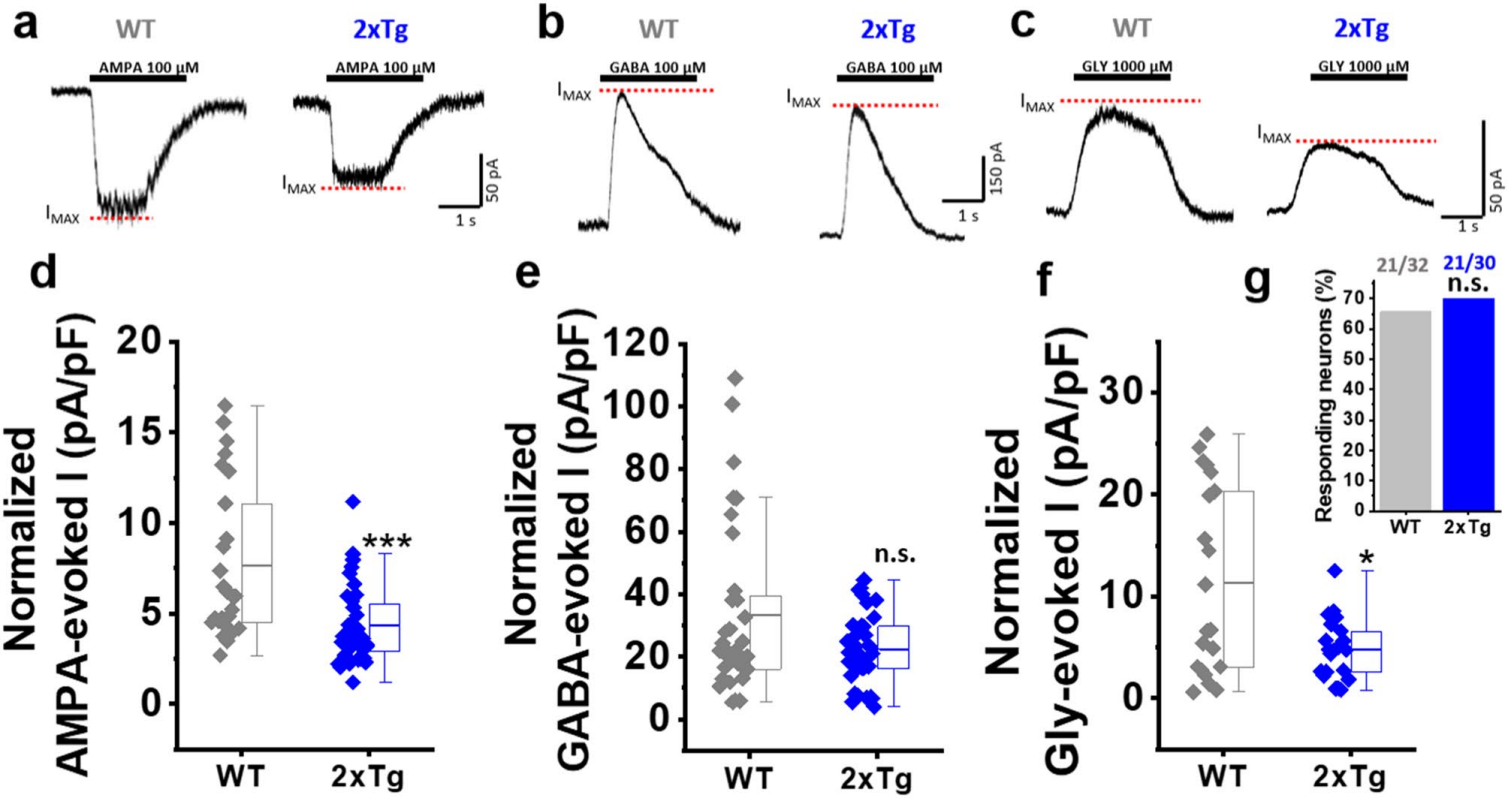
Decreased excitatory and inhibitory post-synaptic receptors in the nAc of 2xTg. (**a-c**) Representative post-synaptic currents evoked by extracellular application of AMPA 100 µM (**a**), GABA 100 µM (**b**) and Glycine (Gly) 1000 µM (**c**) in WT and 2xTg mice. Segmented lines in red indicate the level of the maximum current reached (I_MAX_). (**d-g**) Quantification of the amplitude of the average evoked current (I_MAX_) by AMPA (**d**) (U = 209, z-score = -3.687, p = 1.1E-4), GABA (**e**) (U = 411, z-score = -0.564, p = 0.287) and Gly (**f**) (U = 144, z-score = -1.911, *p* = 0.028), as well as the percentage of neurons responding to Gly 1000 µM (**g**) (χ^2^(1,62) = 0.1356, p = 0.7126). Boxes indicate interquartile range (IQR); center lines, median; whiskers, 1.5 × IQR (interquartile range). * denotes *p* < 0.05, *** *p* < 0.001 (two-tailed Mann–Whitney test for (**d**–**f**) and Chi-square test for (**g**). Responding neurons for AMPA: WT (n = 26) and 2xTg (n = 36). GABA: WT (n = 32) and 2xTg (n = 36). Gly: WT (n = 21) and 2xTg (n = 21). Neurons obtained in at least 4 independent experiments (5 mice for WT and 4 mice for 2xTg).

Next, we analyzed ligand-evoked AMPAergic, GABAergic, and glycinergic currents to confirm if these ionotropic membrane receptors that are present in the nAc were affected in 2xTg mice. We observed a decrease in the maximum evoked current for AMPA and glycine (Gly), but not for GABA. This was expressed as current density (current amplitude divided by cell capacitance; see details in methods Sect. “Current- and Voltage-clamp experiments in dissociated MSNs”). For example, we observed a decrease of ≈ 40% in current density for AMPA-evoked current (WT: 7.62 ± 0.83 vs. 2xTg: 4.34 ± 0.35) (Fig. 4a,d), but no changes were observed for GABA-evoked currents (Fig. 4b,e). On the other hand, the Gly-evoked current density was decreased by ≈ 60% in 2xTg with respect to WT (WT: 11.34 ± 2.01 vs. 2xTg: 4.80 ± 0.65) (Fig. 4c,f). More importantly, the exogenous applications of GABA and AMPA produced responses in all studied neurons (WT and 2xTg). However, the application of a saturating concentration of glycine (1000 μM) was only able to induce currents in 70% of neurons in both WT and 2xTg (Fig. 4g).

### Glycinergic and AMPAergic synaptic activity in the nAc is reduced in the 2xTg mouse model

The presence of AMPA, GABA, and glycinergic synaptic activity in the nAc has been previously reported^30,33–35^. For this reason, and to evaluate if there was any synaptic alteration in the excitatory/inhibitory transmission, we recorded AMPA, GABA, and glycinergic miniature currents in the nAc of WT and 2xTg mice at 6–7 months of age.

**Figure 5:**
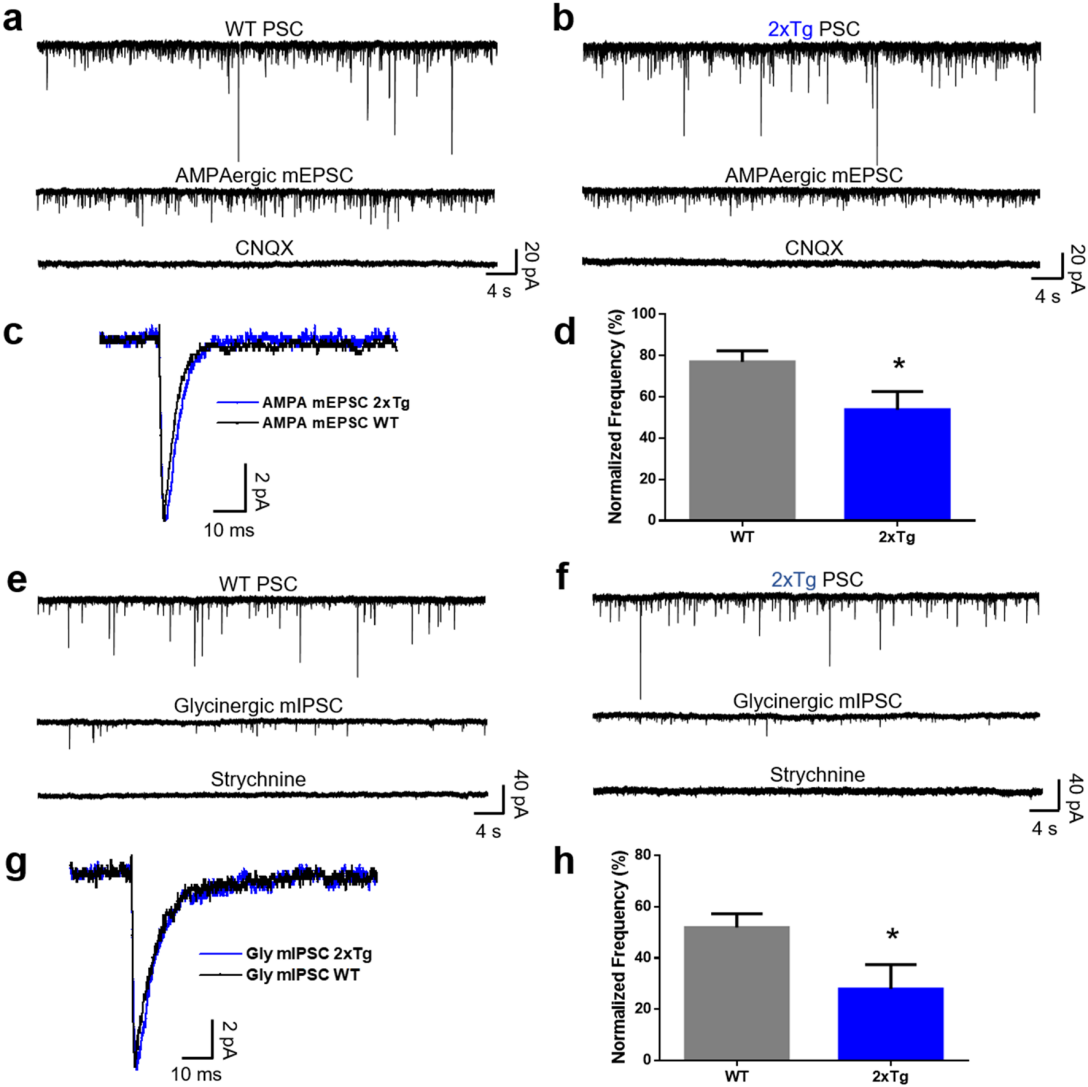
Decreased frequency of AMPAergic and glycinergic synaptic events. (**a,b**) Representative current traces of 1 min duration of synaptic activity in the nAc of WT and 2xTg mice, respectively. The first trace shows the total PSC, the second trace shows the isolated mEPSC mediated by AMPA receptors, and the third trace shows the blockade of the AMPA mEPSC by CNQX. (**c**) Average trace of the AMPA mEPSC in the nAc of WT (black trace) and 2xTg (blue trace) mice. (**d**) Graph shows the reduction of the normalized frequency of AMPAergic events in the 2xTg compared to WT mice (t(16) = 2.233, *p* = 0.0401). (**e,f**) Representative traces of 1 min duration of glycinergic synaptic activity in the nAc of WT and 2xTg mice, respectively. The first trace shows the total PSC, the second shows the isolated mIPSC mediated by GlyR, and the third trace shows the blockade of the glycinergic mIPSC by strychnine. (**g**) Average glycinergic mIPSC in the nAc of WT (black trace) and 2xTg (blue trace) mice. (**h**) Graph shows the reduction of the normalized frequency of glycinergic events in the 2xTg compared to WT mice (t(11) = 2.281, *p* = 0.0435). Data are mean ± SEM, unpaired Student’s t-test for (**d**,**h**), *p < 0.05, n = 9 WT and 9 2xTg for AMPA; n = 13 WT and 9 2xTg for glycine. Neurons obtained from at least 4 independent experiments. Number of mice: WT = 6 and 2xTg = 4.

We found that some properties, such as amplitude and decay time constant, of the AMPA synaptic currents did not differ between both genotypes (Fig. 5a–c). However, when we analyzed the frequency of AMPA currents, after normalizing it to the overall currents in the absence of any antagonist, we found a statistically significant reduction in the 2xTg mice (77 ± 5% in WT and 54 ± 9% in 2xTg) (Fig. 5d). The average AMPAergic trace was similar between genotypes as shown in Fig. 5c. For example, the amplitude of the current was 8.8 ± 0.5 pA in WT and 7.9 ± 0.6 pA in 2xTg mice (Supplementary Fig. 3). Although the decay constant was higher in the 2xTg mice (3.8 ± 0.4 ms in WT versus 5 ± 0.4 ms in 2xTg), these values were not statistically different (Supplementary Fig. 3).

A similar case was found in miniature glycinergic synaptic currents in the nAc of WT and 2xTg mice. Representative traces are shown in Fig. 5e,f, for WT and 2xTg, respectively. The amplitude and decay constant of the currents in WT and 2xTg mice were not different between genotypes (Fig. 5g). For instance, the average amplitude of the traces was 9.9 ± 1 pA in WT and 9.6 ± 0.6 pA in 2xTg (Supplementary Fig. 3), and the decay constant was also similar between the genotypes (Supplementary Fig. 3). However, the quantification of the frequency of glycinergic miniature currents normalized to the total number of events showed a statistically significant decrease from 52 ± 5% in WT nAc to 28 ± 9% in 2xTg (Fig. 5h).

The analysis of GABAergic synaptic events is shown in Supplementary Fig. 4. The data show that GABA_A_ miniature currents were similar in the nAc of WT and 2xTg mice. For example, the normalized frequency was 62 ± 12% in WT and 48 ± 7% in 2xTg. The values for current amplitude (14.2 ± 1.3 pA in WT and 14 ± 0.9 pA in 2xTg) and decay constant (12 ± 1.4 ms and 14 ± 1.6 ms for WT and 2xTg, respectively) did not differ between the mice models (Supplementary Fig. 4).

It is noteworthy that not all neurons in the nAc displayed glycinergic synaptic events. For instance, only 54% (n = 13) of neurons in the WT and 67% (n = 9) of neurons in the nAc of 2xTg mice had glycinergic synaptic events, however, there was no statistical difference between the genotypes. On the other hand, every neuron recorded in the nAc showed AMPA (n = 18) and GABAergic synaptic events (n = 19) in WT and 2xTg mice (Supplementary Fig. 3).

### Reduced presence of presynaptic and postsynaptic markers in the 2xTg mice

**Figure 6:**
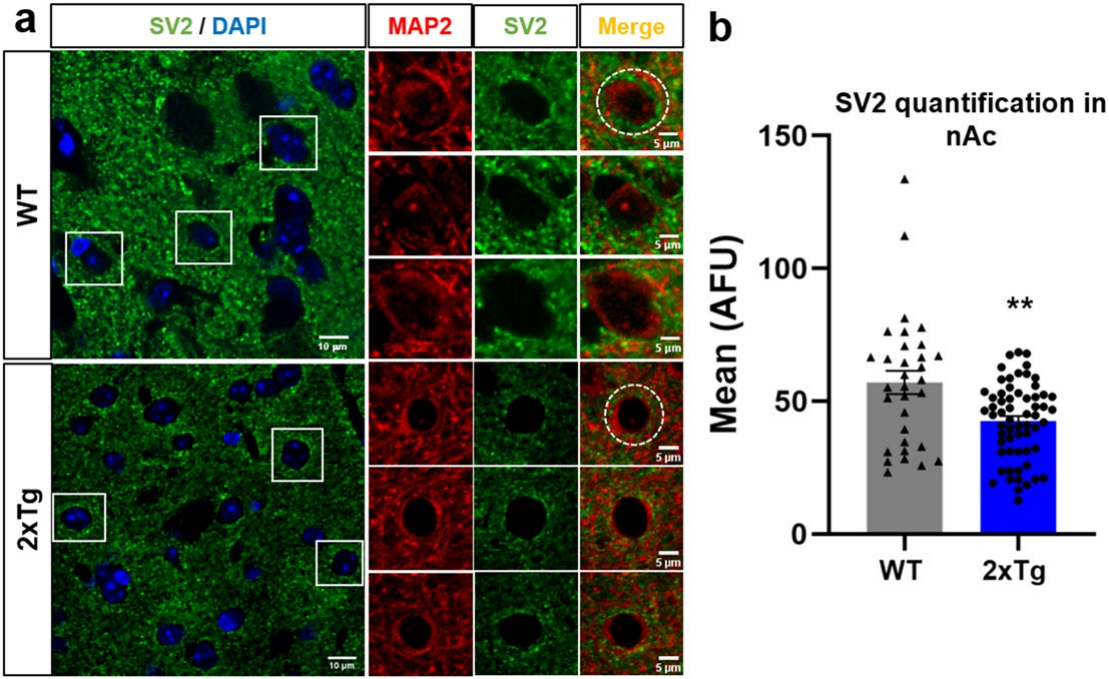
Reduced presence of SV2 in the nAc of 2xTg mice. (**a**) Immunostaining of coronal slices (30 μm) and high magnification images showing SV2 protein in the nAc from WT and 2xTg mice. (**b**) Quantification of SV2 fluorescence intensity in the soma of nAc neurons (dashed circles in (**a**) show regions of interest). Data are mean ± SEM and each data point reflects one soma. Unpaired Student’s t-test (t(90) = 3.243, **p = 0.0017). n = 3 WT and 2xTg mice. Images were analyzed with ‘ImageJ’ 1.8.0_112 (NIH), https://imagej.nih.gov/ij.

A large body of evidence support the existence of synaptic failure in early stages of AD pathology that is well characterized, among other features, by decreased levels of pre- and post-synaptic proteins, as well as synaptic impairment^4,36–39^. Using SV2 (a synaptic vesicle protein) as a pre-synaptic marker, we performed IHC analyses to assess the expression of this protein in the nAc of WT and 2xTg mice. Figure 6a shows coronal slices containing the nAc and a magnified neuron. The data showed that immunofluorescence associated to SV2 was reduced by approximately 20% in the nAc of the 2xTg mice (Fig. 6b).

**Figure 7:**
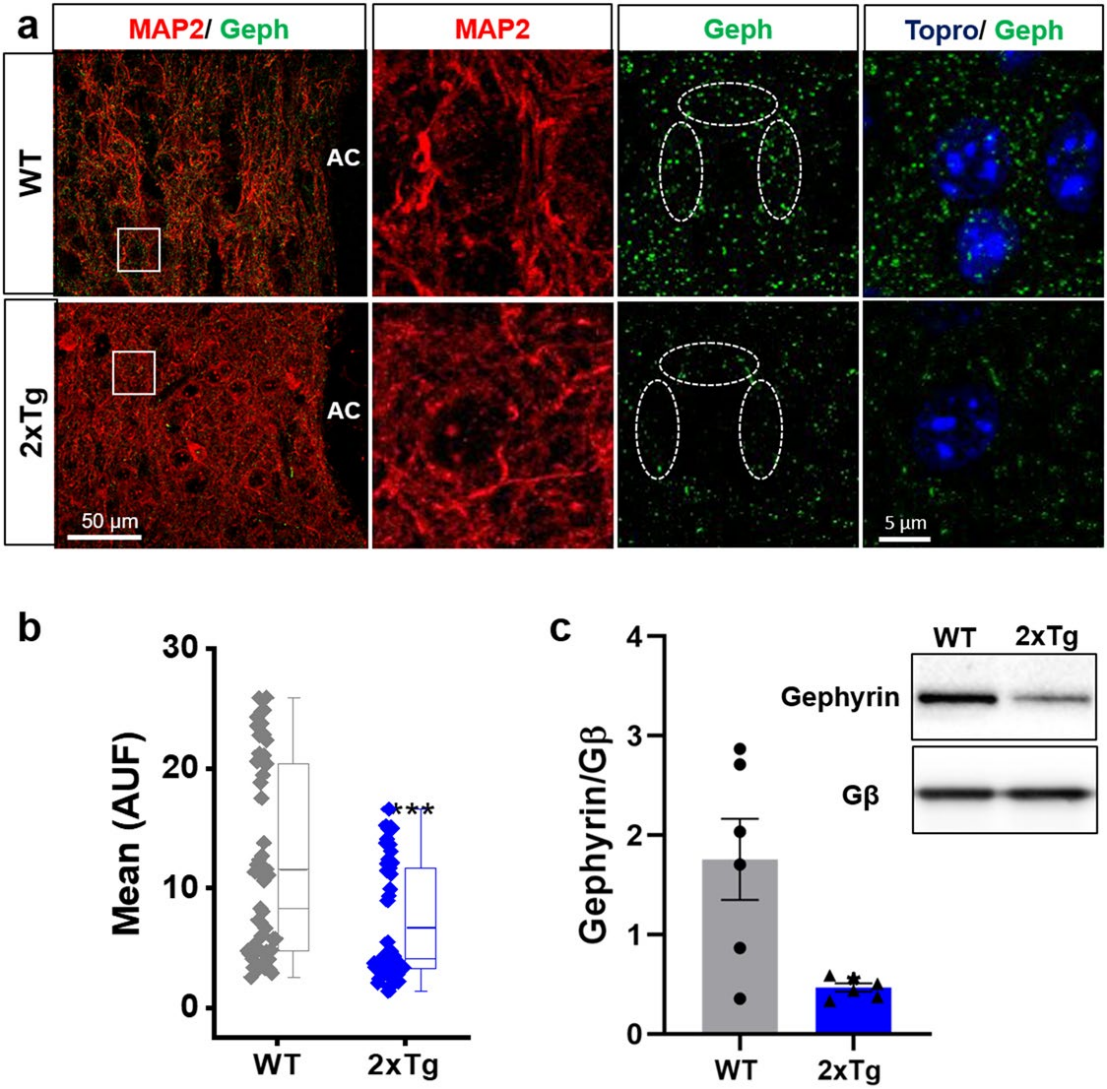
Gephyrin protein expression is reduced in the nAc of 2Tg mice. (**a**) Immunostaining of coronal slices (30 μm) and high magnification images (insets) showing gephyrin protein in the nAc from WT and 2xTg mice. (**b**) Quantification of gephyrin fluorescence intensity in the soma of nAc neurons (dashed circles in (**a**) show regions of interest). In the 2xTg mice, gephyrin fluorescence intensity was significantly decreased. Data are mean ± SEM and each data point reflects one soma (***p < 0.001). (**c**) Western blot of nAc from WT and 2xTg animals for gephyrin and Gβ (loading control). Western blot analysis shows lower levels of gephyrin in 2xTg compared with WT mice (Black lines at the edge of each blot indicates cropping from original full-length blots available in Supplementary Fig. 5). Data are mean ± SEM and each data point reflects one experiment (t(5.112) = 3.146, *p = 0.0247). Mann–Whitney test for (**b**) and Unpaired Student’s t-test with Welch’s correction for (**c**). n = 3 WT and 2xTg mice. Images were analyzed with ‘ImageJ’ 1.8.0_112 (NIH), https://imagej.nih.gov/ij.

The previous results showed that the inhibitory synaptic transmission mediated by glycine was reduced in the nAc of the 2xTg mouse. Therefore, we analyzed the expression of gephyrin, a postsynaptic protein that anchors glycine and GABA_A_R to the synapsis^40^. The data showed that the immunostaining of this protein was markedly reduced in the nAc of the 2xTg mice, and the quantification showed that the reduction was ≈40% (Fig. 7a,b). In agreement with the IHC assay, the western blot analysis of gephyrin also showed a significant reduction in the nAc of 2xTg (Fig. 7c, original full-length blots available in Supplementary Fig. 5 and methodological details in Supplementary Information Sects. 1.3 and 1.4). These results support the idea that both the pre and post-synaptic structures were altered in the 2xTg mice in agreement with the reduced levels in synaptic transmission.

### Reduced level of glycine receptor expression in the nAc of 2xTg

**Figure 8:**
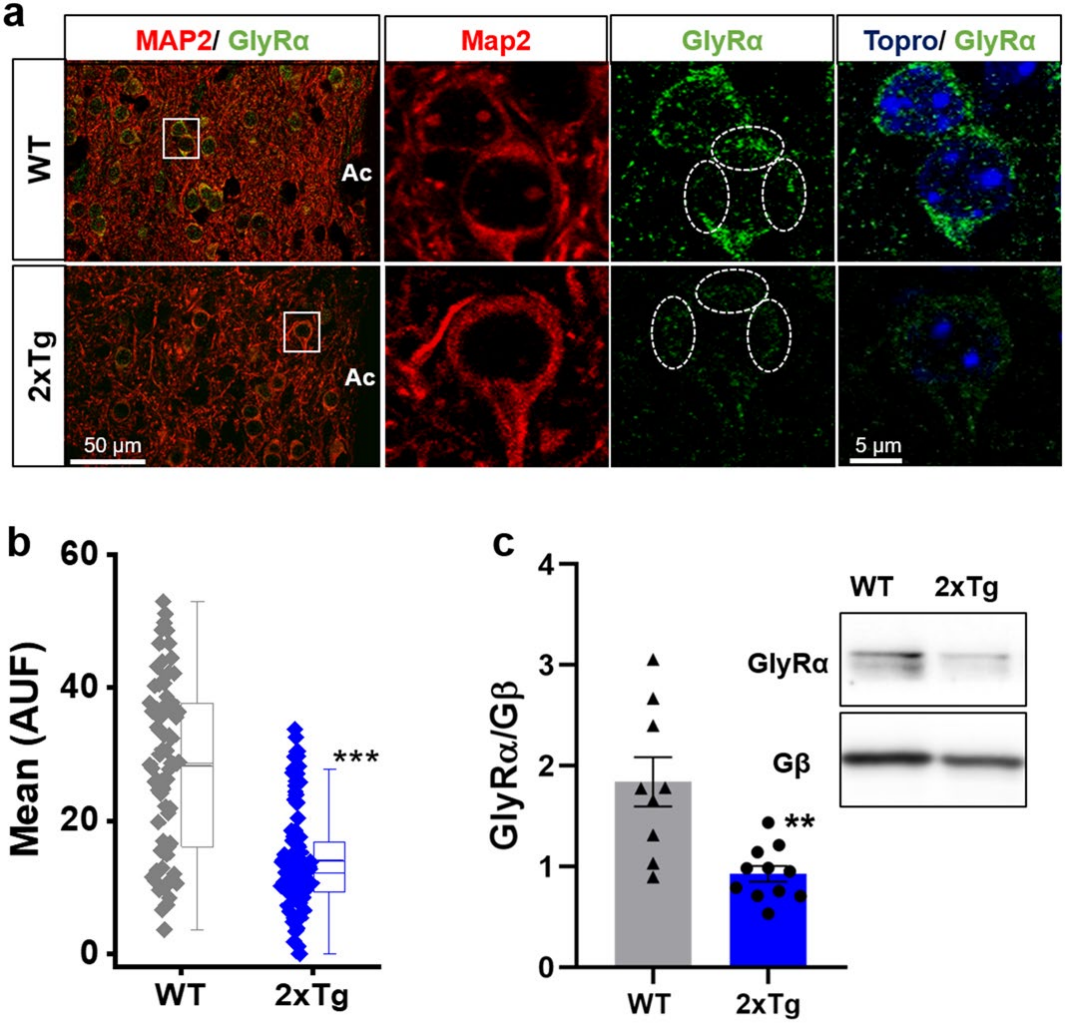
Expression of GlyRα is reduced in the nAc of 2xTg mice. (**a**) Immunostaining of nAc coronal slices (30 μm) and high magnification images (insets) showing GlyRα protein in the nAc from WT and 2xTg mice. (**b**) Quantification of GlyRα fluorescence intensity in the soma of nAc neurons (dashed circles in (**a**) show regions of interest). GlyRα fluorescence intensity was significantly decreased in 2xTg mice. Data are mean ± SEM and each data point reflects one soma (*p* < 0.0001). (**c**) Western blot of nAc from WT and 2xTg animals for GlyRα and Gβ (loading control). Western blot analysis shows lower levels of GlyRα in 2xTg compared with WT mice (Black lines at the edge of each blot indicates cropping from original full-length blots available in Supplementary Fig. 5). Data are mean ± SEM and each data point reflects one experiment (t(9.688) = 3.546, *p* = 0.0056). Mann–Whitney test for (**b**), Unpaired Student’s t-test with Welch’s correction for (**c**). **p < 0.01, ***p < 0.001. n = 4 WT and 2xTg mice. Images were analyzed with ‘ImageJ’ 1.8.0_112 (NIH), https://imagej.nih.gov/ij.

Using an array of biochemical experiments, we have shown that there was an impairment in inhibitory transmission in the nAc of 2xTg compared to WT, especially in terms of glycinergic transmission. Hence, we next evaluated the expression of GlyRs in the nAc using an antibody that recognizes all alpha subunits (pan α antibody) of the receptor. The data from IHC assays (Fig. 8a) showed that the immunostaining of GlyRs was considerably reduced in the 2xTg. This reduction was about 50% when compared to the nAc of WT mice (Fig. 8b). Western blot analysis using the same antibody (Fig. 8c, original full-length blots available in Supplementary Fig. 5) also demonstrated that GlyR protein expression was reduced in the nAc of 2xTg mice.

## Discussion

During the last few decades in AD research, a body of evidence has emerged implicating the hippocampus and cerebral cortex as two main brain regions affected in the pathology, contributing to the understanding of their roles in cognitive and memory dysfunctions^41^. On the other hand, even though basal nuclei also exhibit pathological AD features they have received much less attention. For example, magnetic resonance imaging studies in the striatum have revealed volumetric reduction and significant shape alterations in mild cognitive impairment (MCI)^42^, as well as in AD patients^43,44^. Moreover, accumbal atrophy^45^, as well as the presence of amyloid beta and tau depositions, two widely described markers of AD pathology, has been reported in the pathology^46–48^. In agreement, we detected the presence of Aβ in the nAc in the 2xTg model of AD and although extracellular Aβ deposition was minor, the use of Th-S allowed us to visualize the presence of β-sheet-containing aggregates that could account for the presence of amyloid-related proteins^49^. Similarly, the presence of intraneuronal Aβ was previously reported in the cortex and hippocampus of several transgenic AD mice models^37,50,51^, including the 2xTg mice, and also in human subjects^52,53^. Interestingly, previous studies have shown that intracellular Aβ precedes extracellular accumulation^4,54^ and it is considered to be an early event in AD pathology. Other commonly found pathological features of a well-established AD-like pathology, such as hippocampal astrogliosis^55^ or neuronal loss^56^, were not observed in this mouse model. Thus, the present results indicate that intra neuronal Aβ is likely associated to the alterations in functional properties of MSNs in the nAc.

Using electrophysiological recordings in dissociated neurons and brain slices, we found an increase in neuronal excitability and changes in synaptic transmission in accumbal neurons. In relation to the increased excitability, we found higher rates of action potential (AP) firing in the 2xTg model. Additionally, the rheobase constant of 2xTg was significantly lower than in the WT mice, and a higher percentage of the 2xTg neurons fired AP spontaneously. Collectively, the data support the idea that neurons in nAc exhibit higher excitability at resting conditions, or a predisposition to AP firing. These results are noteworthy and in agreement with the notion that the presence of Aβ increases neuronal excitability^57,58^. For example, the accumulation of Aβ intracellularly in hippocampal^32^ and cortical^59^ neurons was associated to an increase in neuronal excitability during current injection. Similar results were found in the present study in nAc reflected as an increase in firing frequency recorded with and without current injections. In addition, the amplitude and the threshold of the action potentials were altered in the 2xTg mice reflecting alterations on active membrane properties. On the other hand, passive membrane properties, such as membrane potential and input resistance were not affected by the AD pathology. Thus, our study shows that accumbal neurons are hyperexcitable in the AD mice and this data is in agreement to previous reports obtained in other brain regions, such as the hippocampus of murine AD models, and even in human subjects^60,61^. Therefore, to our knowledge, this is the first study that reports an increase in membrane excitability in the nAc of an AD mouse model. Therefore, it is possible to suggest that the nAc, part of the mesolimbic system that is important for motivation, reward, and drug addictive properties, is altered and future studies should look into this possibility.

Several alterations in synaptic properties have been found in AD models at the onset of the disease^62,63^ when Aβ appears as one of the main pathological agents^1,39^. Aβ induces a plethora of synaptic deficits, including a well-established synaptic failure characterized by the downregulation of post-synaptic receptors and neurotransmitter release^39^. In the present study, we found that components of excitatory and inhibitory transmissions in the nAc of 2xTg mice were altered. The activation of AMPA and glycine receptors, but not GABA_A_, showed membrane currents with reduced amplitude and current density. These reductions likely reflect decreases in the number of receptors present in these AD altered neurons. We also evaluated if the reduction in receptor activation was accompanied by changes in post-synaptic receptor function. However, the results did not show any major changes in the amplitude of AMPA and glycine miniature currents nor in the decay time, indicating that the function of these synaptic receptors was not affected, thus suggesting that extra-synaptic receptors might be compromised. In addition, the results showed that the frequency of AMPA- and glycine-mediated miniature currents were decreased in the 2xTg suggesting that the pathology might be affecting pre-synaptic release and/or diminishing the number of release sites in nAc. These results are in agreement with the reduced level of the pre-synaptic protein SV2 that was found in the 6 months old mice. Additionally, the AD mice showed a reduced expression of gephyrin, a post-synaptic scaffolding protein that anchors inhibitory receptors to the post-synapsis^40^. These findings, along with the reduced expression of GlyRs in this AD model, agree with the idea that inhibitory drive is reduced in nAc. However, given that AMPA transmission was also affected, a question of how is it that the decrease in both neurotransmissions (excitatory and inhibitory), with opposite effects, can contribute to an increase in neuronal excitability? Impairment of excitatory and inhibitory synaptic transmission may indeed be involved in the altered neuronal excitability, as previously reported in the hippocampus and cortex of AD models^64–69^. However, it is unclear whether changes in excitatory/inhibitory drive in AD mouse models represents a compensatory mechanism for hyperexcitability, or vice versa. Overall, these synaptic and excitability alterations, together with the presence of intraneuronal Aβ in MSNs, add another brain region to previously reported areas such as the cortex and hippocampus that are related to memory, learning, and cognition, that are affected in the disease^70,71^.

What consequences might these changes in excitability and synaptic function have for nAc function? The nAc is a critical region that integrates information from several brain areas. Its excitability is regulated by intrinsic and extrinsic mechanisms such as excitatory (AMPA) transmission from the PFC, hippocampus, and basolateral amygdala, inhibitory transmission (GABA and glycine) from the midbrain and local collaterals inter-MSNs in the nAc, and dopaminergic control from the VTA^15^. Thus, alterations in excitatory and inhibitory receptors might affect synaptic connectivity with other affected regions in the disease, such as the hippocampus^1^. For example, synaptic plasticity in hippocampal inputs into the nAc is necessary for the formation of reward-related memories. Alterations in the excitability and inputs to the nAc might be related to symptoms of AD because patients exhibit significant apathy and anhedonia^72^. In this regard, a recent study by Nobili et al. showed that outflow of dopamine in the nAc is reduced in another AD mice model resulting in a deterioration in reward processing^73^. Therefore, it is tempting to speculate that nAc-altered physiology could play a critical role in the observed non-cognitive reward-seeking behaviors of AD patients such as motivation, consumption, and even addictive behaviors.

In conclusion, our results provide novel information relating to Aβ pathology in a poorly studied area in the field of AD neuropathology that might have important implications for the establishment and progression of the disease. To date only few studies from animal models and humans are available, thus much information is needed to understand the real dimension of the vulnerability of the ventral striatum in early stages of AD. Finally, the data points to a central role of intracellular Aβ accumulation in the physiological dysfunction in AD.

## Supplementary information

Supplementary Information.

## Data Availability

The datasets used and/or analyzed during the current study are available from the corresponding author on reasonable request.
