# Supplementary material for: Changes in neuronal excitability and synaptic transmission in nucleus accumbens in a transgenic Alzheimer’s disease mouse model
Source: Sci Rep. 2020 Nov 11;10:19606. doi: 10.1038/s41598-020-76456-w (PMC7659319; doi:10.1038/s41598-020-76456-w)
Supplement: Supplementary file 1 — Supplementary Information. [file 41598_2020_76456_MOESM1_ESM.pdf]

Supplementary Information for

Changes in neuronal excitability and synaptic transmission in Nucleus Accumbens in a transgenic Alzheimer's disease mouse model

Fernández-Pérez, E.J.*, Gallegos, S.*, Armijo-Weingart, L., Araya, A., Riffo-Lepe, N.O., Cayuman, F. and Aguayo, LG*.

Laboratory of Neurophysiology, Department of Physiology, Universidad de Concepción, Barrio Universitario s/n P. O. Box 160-C, Concepción, Chile.

*To whom correspondence should be addressed:

Eduardo J. Fernandez-Perez, PhD, Scarlet Gallegos, PhD and Luis G. Aguayo, PhD, Department of Physiology, Universidad de Concepción, P. O. Box 160-C, Concepción, Chile. Tel.: 56-41-203380; Fax: 56-41-245975; emails: edfernandez@udec.cl, scarletgallegos@udec.cl, and laguayo@udec.cl

1. Supplementary Methods

1.1. *Mechano-chemical dissociation of MSNs*. Acute slices containing nAc from 6 months old mice were incubated in oxygenated aCSF (95% O_2/5% CO_2) containing pronase (0.4 mg/mL) for 30 min at 37°C. Then, the nAc was dissected with a scalpel in trituration buffer containing (in mM): NaCl 20, *N*-methyl-d-glucamine (NMG) 130, KCl 2.5, $MgCl_2$ 1, Hepes 10, glucose 10, adjusted to pH 7.4 and 340 mosmol/L and dissociated by mechanical trituration in the recording well (Nunclon™ delta surface, Thermo Scientific, USA) using different pipettes (P1000 and P200, and self-drawn glass-pipette, 10-15 times each). Cells were allowed to settle and stabilize for 20 min on the patch-clamp setup at room temperature (~ 21°C), and the trituration buffer was gradually replaced (1 ml/min) with normal external solution (NES) delivered through a perfusion line that contained (in mM): 150 NaCl, 5.4 KCl, 2.0 $CaCl_2$, 1.0 $MgCl_2$, 10 glucose and 10 HEPES (pH 7.4 adjusted with NaOH, 310 mOsm/L).

1.2. *AP parameters, input resistance and rheobase calculations.* Threshold was numerically estimated from first derivative in a V’ versus V phase space projection. From this value, amplitude was calculated to the maximum value reach by the AP. Finally, we obtained the half width of the AP peak expressed as duration. Input resistance was obtained from the slopes in V/I curves in hyperpolarizing current steps. Rheobase was extrapolated from spikes vs. injected current curves using Origin 2019b (Origin Lab, USA). Spontaneous spike firing frequency was obtained using pClamp10 software (Molecular Devices, USA).

1.3. *Protein extraction.* The nAc was dissected from 300 μm thick slices, and the tissue was triturated in lysis buffer 1x (3.15 mM Ditiotreitol, EDTA 1 mM, protease inhibitor cocktail 1X). The solution was sonicated 3 times for 3 minutes and centrifuged for 10 minutes at 8,000 g. Then the supernatant was stored at -80 °C.

1.4. *Western Blot (WB).* Tissue homogenate (50 μg protein) were subjected to electrophoresis on 10% SDS PAGE gels. Proteins were blotted onto PVDF membranes (Bio-Rad) and blocked with 5% milk in 1X TBS-0.1% Tween 20 for 1 hour with stirring. Membranes were cut according to the expected molecular weight for each of the analyzed proteins. Subsequently, each membrane was separately incubated with primary antibodies for αGlyR (1:1000, rabbit, SySys), GlyR (1:200, rabbit, Alomone), Gephyrin (1:500, mouse, SySys) and anti Gβ (1:1000, rabbit, Santa Cruz) overnight. After washes with 1X TBS and 0.1% Tween 20, membranes were incubated for 2 hours with anti-rabbit and anti-mouse secondary antibodies conjugated to HRP (1:5000, Santa Cruz). The immunoreactivity of the proteins was detected using a chemiluminescence reagent (Promega). Levels of Gβ were used as a loading control. Band intensities were analyzed and compared using 'ImageJ' 1.8.0_112 (NIH), https://imagej.nih.gov/ij.

2. **Supplementary Figures**

Supplementary Figure 1

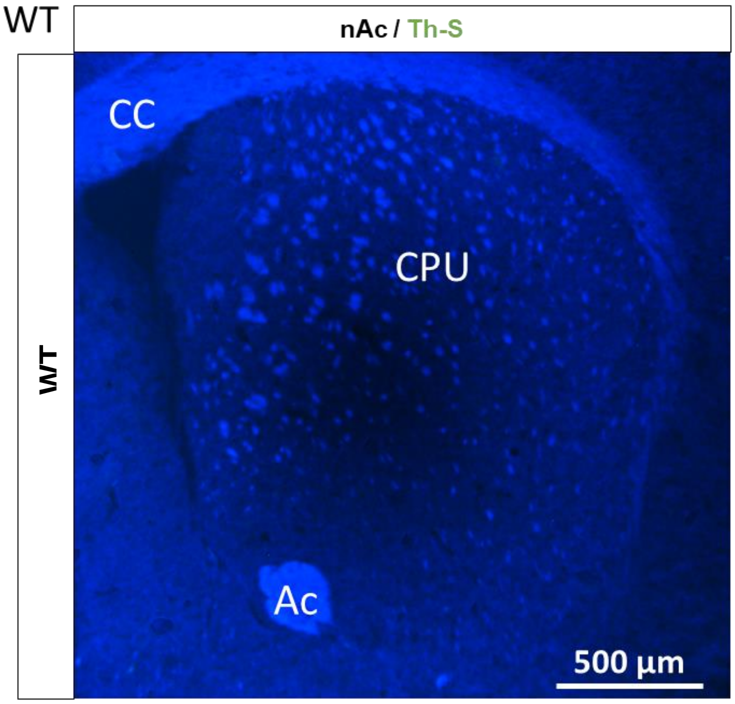

Supplementary Figure 1. Absence of Th-S positive amyloid aggregates and in nAc of WT. Th-S and DAPI staining in nAc of 6 months old WT. CC: corpus callosum; CPU: caudate-putamen; AC: anterior commissure.

Supplementary Figure 2

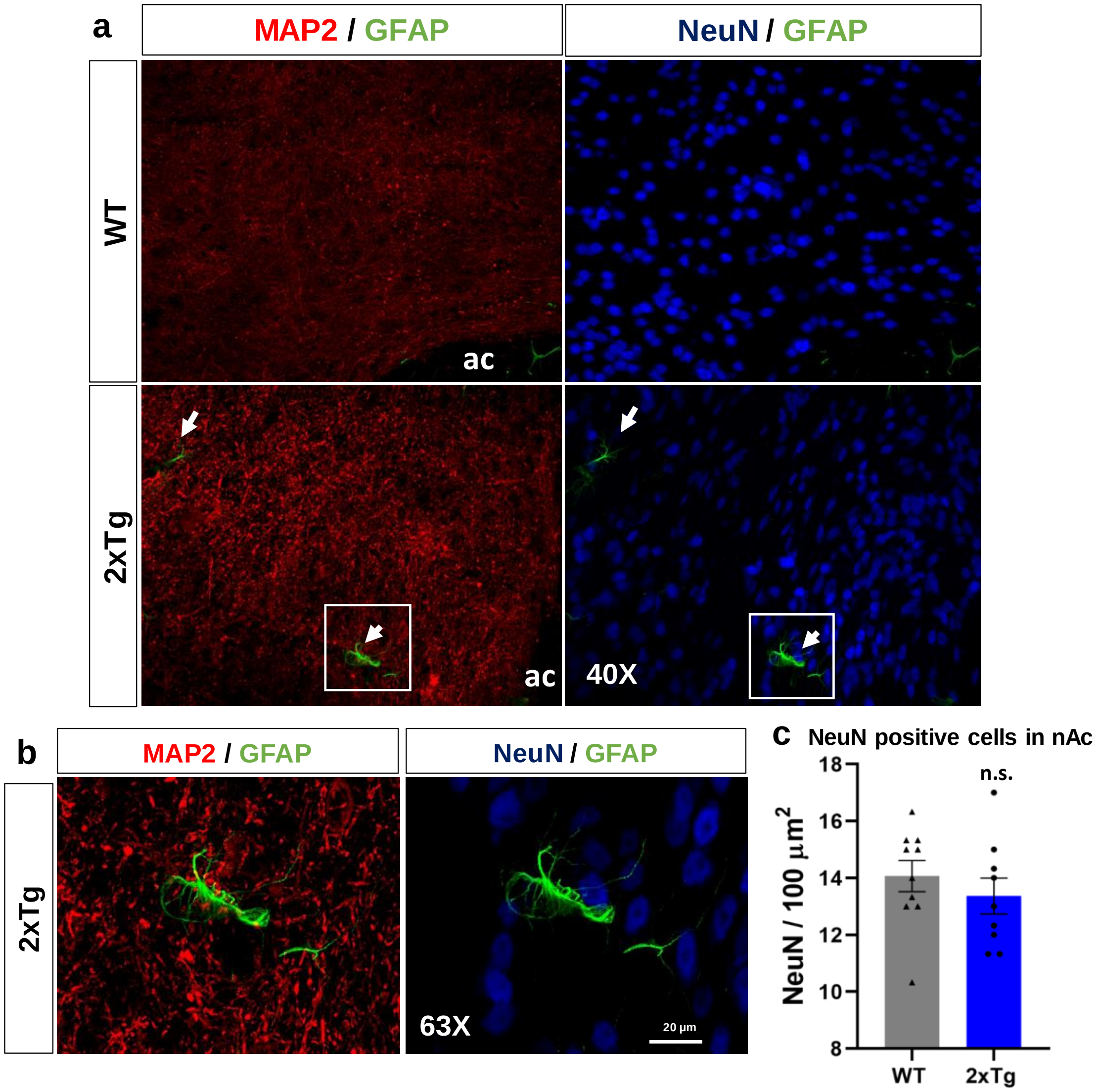

Supplementary Figure 2. Presence of GFAP+ astrocytes in nAc of WT and 2xTg mice. **a,** NeuN and GFAP immunostaining in nAc of 6 months old WT and 2xTg mice. **b,** Higher magnification of square insert of figure **a**, shows astrocytes in nAc of 2xTg mice. **c,** NeuN quantification measured in nAc of 6 months old WT and 2xTg mice. Data are mean ± SEM and each data point reflects one image. ($t(17)= 0.8396$, $p = 0.412$ unpaired Student's t-test). n = 3 WT and 3 2xTg. Images were analyzed with 'ImageJ' 1.8.0_112 (NIH), https://imagej.nih.gov/ij.

Supplementary Figure 3

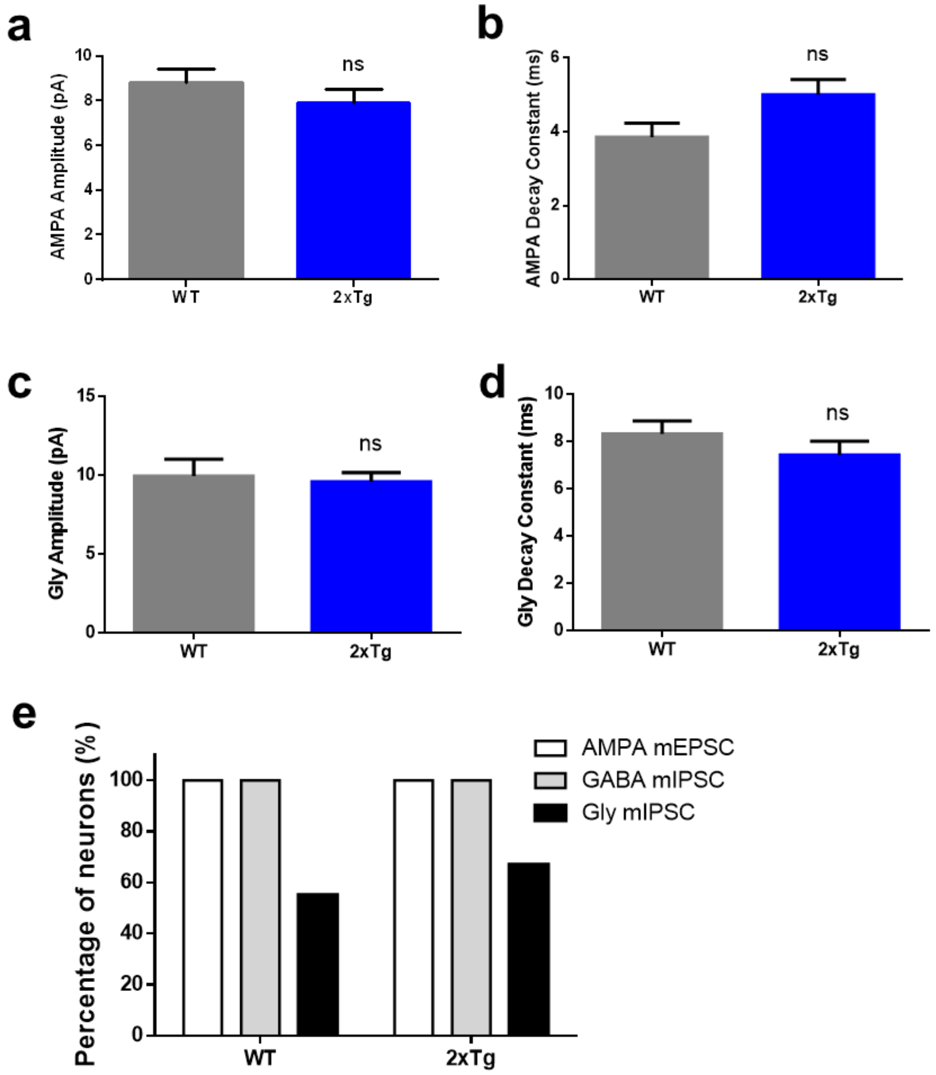

Supplementary Figure 3. Properties of synaptic currents in nAc of WT and 2xTg mice. **a,** Graph shows no difference in the average amplitude of the AMPAergic currents in the nAc of WT and 2xTg mice ($t(16)= 1.069$, $p= 0.3007$). **b,** Graph shows no difference in the average decay constant of the AMPAergic currents in the nAc of WT and 2xTg mice ($t(15)= 2.009$, $p= 0.0629$). **c,** Graph shows no difference in the average amplitude of the glycinergic currents in the nAc of WT and 2xTg mice ($t(10)= 0.2876$, $p= 0.7795$). **d,** Graph shows no difference in the average

decay constant of the glycinergic currents in the nAc of WT and 2xTg mice ($t(10)= 1.112$, $p= 0.2921$). **e,** Graph comparing the percentage of neurons with AMPA, GABA, and glycine synaptic events in neurons of the nAc of WT and 2xTg mice ($X^2= 0.361$, $p= 0.5476$). Data are mean ± SEM, unpaired Student's t-test for **a-d** and Chi square test for **e**, $p> 0.05$, ns. not significant; n= 9 WT and 9 2xTg for AMPA; n= 13 WT and 9 2xTg for glycine; n= 10 WT and 9 2xTg for GABA. Neurons obtained in at least 4 independent experiments. Number of mice: WT = 8 and 2xTg = 6.

Supplementary Figure 4

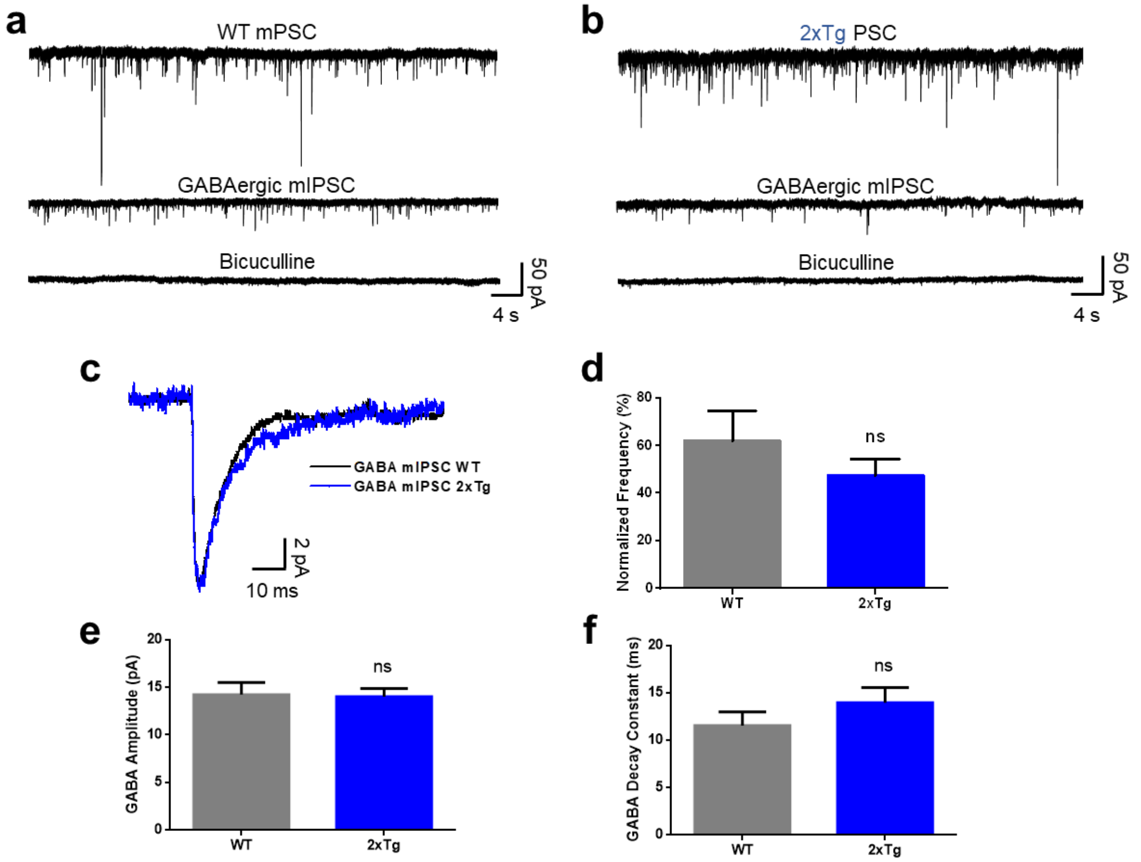

Supplementary Figure 4. GABAergic synaptic events are similar in nAc of WT and 2xTg mice. a, b, Representative traces of 1 minute duration of synaptic activity in nAc of WT and 2xTg mouse, respectively. The first trace shows the total PSC, the second shows the isolated mIPSC mediated by GABA receptors, and the third trace shows the blockade of the GABA mIPSC by bicuculline. **c,** Average trace of the GABA mIPSC in the nAc of WT (black trace) and 2xTg (blue trace) mice. **d,** Graph shows the average normalized frequency of GABAergic events in the 2xTg and WT mice ($t(17)= 1.114$, $p= 0.2807$). **e,** Graph shows no difference in the average amplitude of the GABAergic currents in the nAc of WT and 2xTg mice ($t(17)= 0.1349$, $p= 0.8943$). **f,** Graph shows no difference in the average decay constant of the

GABAergic currents in the nAc of WT and 2xTg mice ($t(17)= 1.103$, $p= 0.2852$). Data are mean ± SEM, unpaired Student's t-test for **d-f**, $p> 0.05$, ns. not significant; n= 10 WT and 9 2xTg. Neurons obtained in at least 4 independent experiments. Number of mice: WT = 3 and 2xTg = 3

Supplementary Figure 5

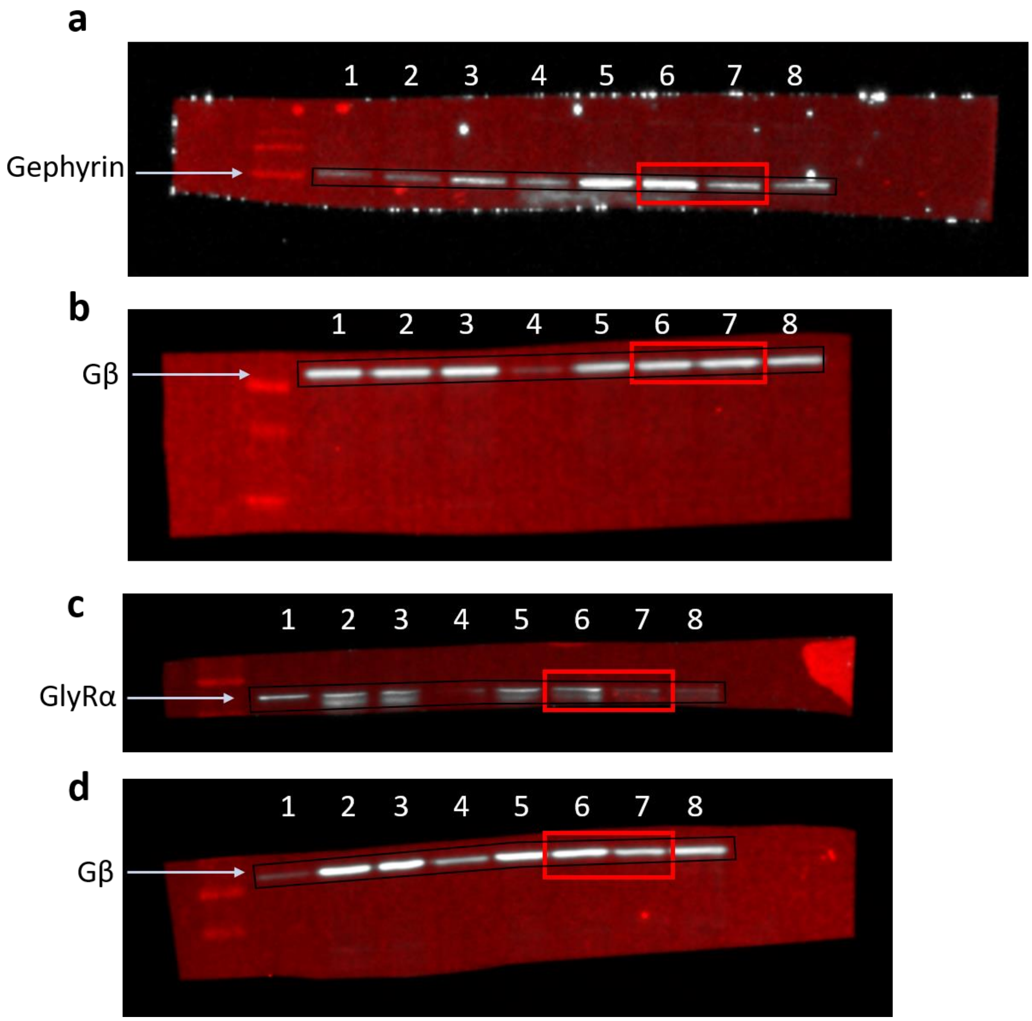

Supplementary Figure 5. Western blot of nAc from WT and 2xTg animals for gephyrin and GlyRα. a–d, Original full-length Western blot showing protein levels of gephyrin (**a**) and GlyRα (**c**) in WT mice and 2xTg (in duplicate for each condition). Gβ was used as loading control (**b** and **d**). From left to right lines are labeled as follows: 1-2 for WT 2 months, 3-4 for WT 12 months, 5-6 for WT 6 months and 7-8 for 2xTg 6 months (2xTg 6M). Lines 6 and 7 from **a** and **b** were used in Figure 7 and lines 6-7 from blots in **c** and **d** were used in Figure 8 (indicated in a red box). Edges of the used blots were outlined with solid black lines.
